# Frailty in middle-aged and older adult postoperative patients with gynecological malignancies structural equation modeling

**DOI:** 10.3389/fpubh.2024.1431048

**Published:** 2024-09-26

**Authors:** Shuo Man, Xiaofang Wu, HaoWen Huang, Jinjin Yu, Ling Xia

**Affiliations:** ^1^Department of Obstetrics and Gynecology, Affiliated Hospital of Jiangnan University, Wuxi, Jiangsu, China; ^2^Jiangnan University, Wuxi, Jiangsu, China

**Keywords:** frailty, gynecological malignancy, self-management, quality of life, structural equation modeling

## Abstract

**Background:**

Frailty and self-management are important determinants of quality of life in cancer patients. However, their synergistic effects and potential mechanisms on quality of life in middle-aged and older adult postoperative gynecologic malignancy patients have not been adequately studied.

**Objective:**

This cross-sectional study aimed to explore the relationship between frailty, self-management, and quality of life in middle-aged and older adult postoperative gynecologic malignancy patients.

**Methods:**

A cross-sectional study was conducted from January 2024 to April 2024 in three gynecological wards of a tertiary hospital in Wuxi. The study recruited 177 patients aged 45 years or older who underwent surgery for gynecologic malignancies (cervical, ovarian, and endometrial cancer). Data were collected using demographic and clinical characteristics, the Edmonton Frailty Scale, the Self-Management Competence Scale, and the EORTC Core Quality of Life Questionnaire. Structural equation modeling was used to explore the interactions between frailty, self-management, and quality of life.

**Results:**

The prevalence of frailty in middle-aged and older adult postoperative gynecologic malignancy patients was 39.5%, with a mean total self-management score of 125.81 ± 13.21 and a mean total quality of life score of 69.26 ± 10.88. The fit indices of the model indicated a good fit, and that frailty had multiple effects on quality of life; specifically, frailty could affect the quality of life directly or through self-management, i.e., self-management partially mediated frailty and quality of life.

**Conclusion:**

Self-management is a mediating variable between frailty and quality of life, suggesting that clinical workers can intervene in self-management skills to improve patient’s quality of life and physical and mental health.

## Highlights

- We presented the current status of frailty, self-management, and quality of life in middle-aged and older adult patients with postoperative gynecological malignancies and their influencing factors, which can be used to improve the quality of life of the patients from different perspectives and to provide a theoretical basis for the clinical implementation of nursing interventions.
- Structural equation modeling clarified the mechanism of action of frailty and quality of life, analyzed in depth the path relationships between the variables, and is innovative in terms of research methodology, which has not yet been reported.

## Introduction

Gynecological cancer is the leading cause of cancer-related deaths in women worldwide (1). The 2022 China Cancer Statistics Report showed that (2) cervical, ovarian, and endometrial cancers are the most common gynecologic malignancies, accounting for 23.6% of female cancers. The overall trend is increasing year by year and towards younger age. Surgery is the preferred option for the treatment of early-stage gynecologic malignancies (3), which usually involves total hysterectomy and extensive lymph node dissection. This type of major surgery often requires a protracted postoperative recovery time. If recovery is slow, it may cause muscle atrophy and loss of fitness, all of which may manifest as frailty.

“Frailty” is an impairment of an individual’s ability to maintain homeostasis following a stressor, as described by an overall decline in strength, endurance, and physiological functioning. This condition increases an individual’s vulnerability to risk of death (4). Frailty reflects biological and phenotypic age, not actual age (5). Recent studies (6) in China have shown a high prevalence of frailty in women aged 45–79, and that frailty in middle-aged and older adults is associated with an increased risk of death and lower life expectancy.

Studies have shown that up to 60% of patients with gynecologic malignancies suffer from frailty (7). Surgery, as a powerful stress factor, may lead to frailty in patients with gynecologic malignancies. This frailty is a direct result of surgical and therapeutic stress. It may occur at a faster rate and to a more severe degree, often occurring during the postoperative recovery period. This frailty is characterized by acute physical and psychological responses such as muscle and physical decay due to slow recovery, pain and fatigue from the inflammatory response caused by surgery, and complications such as infection, hemorrhage, or lymphedema. In addition, malnutrition due to loss of appetite, as well as persistent fear and anxiety about disease recurrence, combine to drive the progression of the frailty state (8).

In gynecologic oncology, frailty has significant negative effects on multiple key indicators of healthcare, which include prolonged hospital stays, increased unplanned readmissions and readmission mortality, and increased incidence of postoperative complications (7, 9). Currently, these findings are primarily based on data from older adult patients, and frailty after surgery for gynecologic malignancies in other age groups has not been adequately studied. The management assessment of patients with gynecologic malignancies should not be based on actual age alone but instead, adopt a comprehensive multidimensional methodology (10–12). Therefore, exploring how to effectively manage the frailty of middle-aged and older adult postoperative gynecologic malignancy patients is of great significance in enhancing their quality of life.

Quality of life is a multidimensional concept constructed on the basis of a specific cultural value system, reflecting the perception and experience of individuals in different cultures and value systems in the pursuit of their respective goals and expectations (13). Studies have confirmed that quality of life is an independent predictor of the health status and prognosis of cancer patients (14). It is one of the most important indicators for evaluating the effectiveness of treatment and the quality of care for patients (15). One study showed that gynecologic cancer patients had a lower quality of life (16). It is essential to focus on the subjective experience and health needs of patients with gynecologic malignancies to improve their quality of life. Although a negative association between frailty and quality of life has been found (17, 18), few studies have involved patients with gynecologic malignancies. The study of the impact of frailty on quality of life in patients with gynecologic malignancies needs to be further expanded.

However, individuals with gynecologic malignancies cannot maintain a healthy quality of life without their own active coping. Self-management is one way of active coping. Self-management is the process by which individuals actively regulate their behavioral, emotional, and physiological responses according to their health needs in order to enhance self-efficacy and health (19). Investigative studies have shown that self-management is closely related to the occurrence of frailty and that patients with poor self-management skills have a more severe degree of frailty (20). This finding has been confirmed in patients with chronic diseases such as hypertension and heart failure (21). One study found that the self-management skills of cervical cancer patients need to be improved (22). As gynecologic malignancies are chronic diseases that require long-term management, maintaining good self-management is essential to improving quality of life. Previous research has shown that high levels of self-management can enhance a patient’s ability to adopt specific strategies to cope with cancer and its treatment, improving disease control and quality of life (23). The implementation of self-management interventions has been reported to positively impact the clinical treatment, psychosocial, and economic outcomes of cancer patients, which include enhanced quality of life, promotion of physical and mental health, and more efficient use of healthcare resources. Supportive self-management interventions, in particular, can achieve such effects (24).

**Figure 1:**
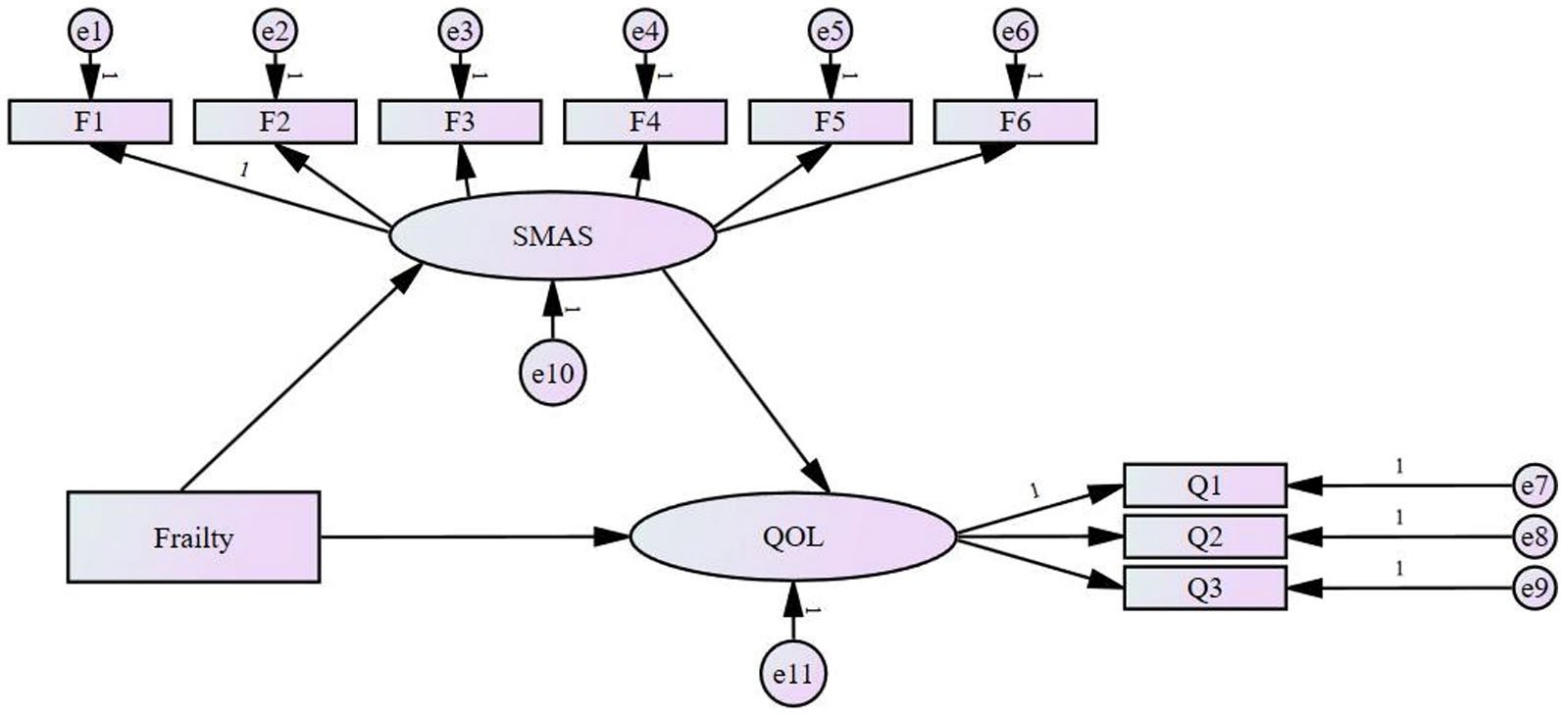
The hypothetical model. SMAS, Self-management Assessment Scale; QOL, quality of life.

In summary, frailty is a growing concern in the global medical community, especially in conditions such as malignancy and cognitive impairment. However, in China, frailty in patients with gynecologic malignancies has not been adequately studied. Frailty and self-management are key factors affecting an individual’s quality of life. Although frailty has been demonstrated to be significantly associated with quality of life (17, 18), no study has yet explored the possibility of self-management as a mediating variable, especially in the group of postoperative gynecologic malignancy patients. We hypothesize that self-management is a potential mediator of frailty and quality of life. Structural equation modeling (SEM) is a statistical method used to analyze the relationship between latent and directly observed variables (25). Therefore, this study used structural equation modeling to explore how frailty improves patients’ quality of life through self-management. The hypothetical model is shown in Figure 1.

## Materials and methods

### Study design and participants

A cross-sectional design was conducted. From January 2024 to April 2024, patients with gynecologic malignancies were continuously recruited from three gynecology departments of a tertiary hospital in Wuxi. The inclusion criteria were (1) diagnosed with cervical cancer, endometrial cancer, or ovarian cancer and undergoing standardized surgical treatment (26–29), (2) aged 45 or above (30), (3) having clear consciousness. The exclusion criteria were patients (1) combined with other malignant tumors, (2) combined with organ failure or life-threatening severe conditions, (3) Patients who have received preoperative pelvic or abdominal radiotherapy or chemotherapy, (4) Patients diagnosed with psychological, mental illness, and language dysfunction (31).

### Ethical considerations

All study subjects were provided written informed consent. The study was approved by the Ethics Committee of the Affiliated Hospital of Jiangnan University (LS2023067), which confirmed that all research was conducted in accordance with relevant guidelines/regulations and that informed consent was obtained from all participants and/or their legal guardians. The study was conducted in accordance with the Declaration of Helsinki.

### General information questionnaire

This questionnaire included demographic and clinical characteristics. Demographics included age, BMI, family history, marital status, menopausal status, parity, education, work status, monthly household income, and primary caregiver. Clinical characteristics included disease type and clinical stage, type of surgery, comorbidity, and medication use. Clinical characteristics were obtained from patient cases.

### Edmonton frailty scale

The Edmonton Frailty Scale (EFS) was used to measure participants’ frailty. It was developed by Rolfson et al. at the University of Alberta in Edmonton, Canada (32). It was translated into Chinese by Xiaohong (33). The Chinese version of the EFS is composed of 11 questions that analyze nine dimensions (health status, independent living ability, social support, medication, nutrition, emotion, incontinence, cognition, and activity ability). The total score of the EFS is 17, with higher scores representing more severe frailty. Based on the scoring guidelines provided on the official website of the Edmonton Frailty Scale, patients were categorized into five grades: 0–3 points for no frailty, 4–5 points for vulnerable, 6–7 points for mild frailty, 8–9 points for moderate frailty, ≥10 points for severe frailty (32). The Cronbach’s alpha coefficient of the Chinese version of the EFS was 0.599 (33). Referring to the relevant literature and considering the patients’ postoperative physical recovery, the Chinese version of the EFS scale was applied in our study to investigate the patients’ postoperative frailty within 5–7 days after surgery (34, 35).

### Self-management assessment scale

The Self-Management Assessment Scale (SMAS) for Cancer Patients developed by Cheng was used to measure the level of self-management among Chinese cancer patients (36). The SMAS consists of six dimensions: daily life management, symptom management, psychological management, communication with medical staff, information management, and self-efficacy. The scale employs a 5-point Likert-type scale (1 = “not important” to 5 = “very important”). The higher the scale score, the better the patient’s self-management ability. According to Cheng, Cronbach’s alpha coefficient of the scale was 0.959 (36). In our study, the Cronbach’s alpha coefficient was 0.962, indicating high internal consistency and reliability of the scale. The scale provides a valid tool for assessing the self-management needs of Chinese gynecologic cancer patients (37). The researchers investigated patients’ postoperative self-management abilities within 5–7 days after surgery.

### EORTC Core Quality of Life Questionnaire

The EORTC Core Quality of Life Questionnaire (EORTC QLQ-C30) is an instrument developed by the European Organization for Research and Treatment of Cancer (EORTC) to assess the quality of life of cancer patients. It was translated into Chinese by Chonghua et al. (38), the Chinese version of the QLQ-C30 was used in this study, which consists of five functional domains, three symptom domains, six single-item measures, and a global Quality Of Life (QOL) scale. All entries were scored on a 4-point Likert scale (1 = not at all, 2 = somewhat, 3 = quite a bit, 4 = very much) except for the global Quality Of Life scale, which was scored on a 1–7 scale. All scores were standardized and converted on a 0–100 scale, with high scores on functioning and global Quality Of Life indicating good status and high scores on symptoms indicating severe problems. The Cronbach’s alpha coefficient of the Chinese version of the QLQ-C30 was 0.74–0.87. The Chinese version of the QLQ-C30 has been validated for Chinese patients with various types of cancer (39). The researchers investigated patients’ postoperative quality of life within 5–7 days after surgery.

### Statistical methods

Data were initially organized using Epidata version 3.02 and then imported into SPSS software version 26.0 for statistical analysis. The overall data were analyzed descriptively, and descriptive data were expressed as mean and standard deviation. Pearson correlation analysis was used to analyze the correlation between frailty, self-management and quality of life. Variance analysis and independent sample t-test were used to analyze the significance of general information on the scores of each variable in the model. The variables in the model were assigned, and the structural equation modeling was performed using IBM SPSS AMOS 28.0. The structural equation modeling variable assignments are shown in Table 1. Indicators such as the chi-square to the degree of freedom ratio (χ^2^/df), the goodness-of-fit index (GFI), the adjusted goodness-of-fit index (AGFI), the normed fit index (NFI), the comparative fit index (CFI), and the root mean square error of approximation (RMSEA) were selected to evaluate the goodness of fit of the model. Combined with the MI index, the covariance relationship was established to adjust the model. The significance of the mediating effect was verified by a bootstrap test using Model 4 in the SPSS software, with 2000 repeated samples. If the 95% confidence interval of the effect did not include zero, the mediating effect was considered significant. In all analyses, a *p*-value of less than 0.05 was considered to indicate statistical significance.

**Table 1:** Structural equation model variable assignment.

Variables	Assignment criteria
Daily life management	F1
Symptom management	F2
Psychological management	F3
Communicate with medical staff	F4
Information management	F5
Self-efficacy	F6
Global QOL scale	Q1
Functioning scales	Q2
Symptom scales	Q3

## Results

### Sample characteristics and its difference among variable scores in the model

A total of 177 patients had a mean age of 58.88 ± 11.04 years, with the highest percentage being 45–59 years old (54.2%), followed by 60–69 years old (27.1%); the vast majority were married (95.5%), had no family history of cancer (87%); the majority of the participants had a BMI of 18.5–24 (48.6%), were menopausal (68.9%), had a history of 1-time birth (62.7%), junior high school education (45.2%), their caregivers were their spouses (67.8%), and retired (37.9%); nearly half of the participants had a household income of ≥5,000 RMB; cervical cancer (41.8%) was more prevalent among the types of disease, followed by ovarian cancer (39.5%); and the clinical stages were ovarian cancer stage II (34.5%) and cervical cancer stage IIA (22.0%); surgery was mostly open surgery (91%); comorbidities were present in 7.4% of patients, including hypertension (5.1%) and diabetes mellitus (2.3%); and medications were used in 5.1% of patients, including anti-hypertensive medicines (4%) and anti-diabetics medicines (1.1%). Age, menopausal status, parity, education, work status, monthly family income, primary caregiver, comorbidity, and medication use were significantly different in frailty score, self-management score, and quality of life score. Family history, marital status, and Type of surgery were statistically different in frailty scores. Disease type and Type of surgery were statistically different in self-management scores. General information about the participants and their differences in scores on each variable are shown in Table 2.

**Table 2:** Relationship among general information and variable scores (*n* = 177).

Variables	n (%)	Frailty	SMAS	QOL
Age, y				
45–59	96 (54.2)	3.85 ± 1.21	134.30 ± 7.55	74.74 ± 9.32
60–69	48 (27.1)	6.10 ± 1.72	121.35 ± 9.08	67.53 ± 6.01
70–79	25 (14.1)	8.40 ± 1.83	111.0 ± 7.33	56.67 ± 7.98
≥80	8 (4.5)	10.88 ± 0.83	97.00 ± 4.66	53.13 ± 6.20
t/F		184.452^c^	107.573^b^	46.845^c^
P		<0.001^**^	<0.001^**^	<0.001^**^
BMI, kg/m^2^				
<18.5	7 (4)	5.57 ± 2.82	125.86 ± 17.95	72.62 ± 13.36
18.5–24	86 (48.6)	5.42 ± 2.56	126.31 ± 14.11	68.99 ± 10.28
>24	84 (47.5)	5.42 ± 2.39	125.30 ± 11.93	69.25 ± 11.35
t/F		0.013^b^	0.125^b^	0.357^b^
P		0.987	0.883	0.7
Family history				
Yes	23 (13)	4.57 ± 1.44	130.78 ± 12.71	73.19 ± 8.69
No	154 (87)	5.55 ± 2.57	125.07 ± 13.16	68.67 ± 11.07
t/F		-2.703^a^	1.949^a^	1.872^a^
P		<0.01^*^	0.053	0.063
Marital status				
Married	169 (95.5)	5.48 ± 2.48	125.79 ± 13.27	68.93 ± 10.90
Divorced	6 (3.4)	3.50 ± 0.55	131.83 ± 7.41	76.39 ± 8.19
Widowed	2 (1.1)	7.50 ± 3.54	110.00 ± 11.31	75.00 ± 11.79
t/F		18.214^c^	2.081^b^	1.654^b^
P		0.032^*^	0.128	0.194
Menopausal status				
Yes	122 (68.9)	6.11 ± 2.57	121.80 ± 13.41	68.93 ± 10.90
No	55 (31.1)	3.89 ± 1.33	134.73 ± 6.99	76.39 ± 8.19
t/F		7.581^a^	-8.418^c^	-9.207^a^
P		<0.001^**^	<0.001^**^	<0.001^**^
Parity, times				
0	5 (2.8)	6.60 ± 1.14	117.40 ± 9.48	70.00 ± 7.45
1	111 (62.7)	4.37 ± 1.78	132.48 ± 9.29	73.87 ± 9.57
2	61 (34.5)	7.25 ± 2.56	114.38 ± 11.25	60.79 ± 7.96
t/F		33.265^c^	66.083^b^	43.517^c^
P		<0.001^**^	<0.001^**^	<0.001^**^
Educational level				
Primary school or less	58 (32.8)	6.95 ± 2.64	114.28 ± 11.18	62.64 ± 8.86
Middle school	80 (45.2)	5.03 ± 2.16	128.33 ± 9.71	70.10 ± 9.16
High school/special secondary	27 (15.3)	4.15 ± 1.56	136.81 ± 8.73	77.78 ± 11.79
College or higher	12 (6.8)	3.58 ± 1.31	140.08 ± 4.60	76.39 ± 9.29
t/F		17.366^c^	62.103^c^	19.016^b^
P		<0.001^**^	<0.001^**^	<0.001^**^
Work states				
Employed	47 (26.6)	3.68 ± 1.29	135.83 ± 6.35	79.08 ± 9.17
Retired	67 (37.9)	5.54 ± 2.12	126.64 ± 11.16	66.92 ± 8.70
Unemployed	44 (24.9)	6.75 ± 2.99	116.36 ± 14.46	64.77 ± 10.46
Peasants	19 (10.7)	6.26 ± 2.21	120.00 ± 11.35	63.60 ± 6.92
t/F		22.898^c^	32.428^c^	25.548^b^
P		<0.001^**^	<0.001^**^	<0.001^**^
Monthly household income, RMB				
1,001–3,000	53 (29.9)	6.64 ± 2.84	117.34 ± 13.92	64.78 ± 10.03
3,001–5,000	41 (23.2)	6.27 ± 2.24	120.61 ± 11.04	64.43 ± 9.13
≥5,000	83 (46.9)	4.23 ± 1.68	133.80 ± 8.24	74.50 ± 9.86
t/F		23.915^c^	44.137^c^	22.617^b^
P		<0.001^**^	<0.001^**^	<0.001^**^
Primary caregivers				
Spouse	120 (67.8)	4.71 ± 1.97	129.57 ± 11.09	71.53 ± 10.05
Children	40 (22.6)	7.43 ± 2.51	115.78 ± 13.22	61.25 ± 8.96
Other caregivers	17 (9.6)	5.76 ± 3.03	122.94 ± 14.61	72.06 ± 12.13
t/F		19.372^c^	20.513^b^	16.479^b^
P		<0.001^**^	<0.001^**^	<0.001^**^
Disease type				
Cervical cancer	74 (41.8)	5.07 ± 2.42	126.61 ± 13.15	70.61 ± 11.31
Ovarian cancer	70 (39.5)	5.86 ± 2.74	123.01 ± 13.50	67.62 ± 10.48
Endometrial carcinoma	33 (18.6)	5.30 ± 1.90	129.97 ± 11.66	69.70 ± 10.59
t/F		1.896^b^	3.430^b^	1.398^b^
P		0.153	0.035^*^	0.25
Clinical stage				
Cervical cancerIA	2 (1.1)	4.00 ± 1.41	141.00 ± 12.73	83.33 ± 0.00
Cervical cancerIB	30 (16.9)	5.53 ± 2.66	123.57 ± 12.69	69.72 ± 11.47
Cervical cancerIIA	39 (22.0)	4.79 ± 2.32	128.90 ± 13.39	70.73 ± 11.92
Endometrial carcinomaI	4 (2.3)	4.25 ± 2.50	141.00 ± 12.73	68.75 ± 4.17
Endometrial carcinomaII	41 (23.2)	5.61 ± 2.25	123.57 ± 12.69	68.70 ± 11.15
Ovarian cancerII	61 (34.5)	5.77 ± 2.62	128.90 ± 13.39	68.03 ± 10.00
t/F		1.116^b^	2.494^b^	1.001^b^
P		0.354	0.330	0.419
Type of surgery				
Laparotomy	161(91)	5.54 ± 2.48	125.02 ± 13.03	68.94 ± 10.98
Laparoscopy	16(9)	4.25 ± 2.21	133.75 ± 12.82	72.40 ± 9.49
t/F		-2.005^a^	2.559^a^	1.212^a^
P		0.047^*^	0.011^*^	0.227
Comorbidity				
Hypertension	9 (5.1)	8.22 ± 2.49	113.33 ± 11.36	56.48 ± 8.10
Diabetes mellitus	4(2.3)	8.00 ± 2.94	113.25 ± 9.98	66.67 ± 11.79
No	164(92.7)	5.21 ± 2.35	127.52 ± 11.91	70.02 ± 10.60
t/F		9.348^b^	6.692^b^	7.197^b^
P		<0.001^**^	0.002^**^	<0.001^**^
Medication use				
Anti-hypertensive	7(4.0)	8.71 ± 2.21	109.14 ± 8.86	54.76 ± 8.13
Anti-diabetics	2(1.1)	8.00 ± 2.83	117.00 ± 15.57	70.83 ± 17.68
No	168(94.9)	5.26 ± 2.38	126.61 ± 12.90	69.84 ± 10.55
t/F		8.281^b^	6.738^b^	6.913^b^
P		<0.001^**^	0.002^**^	<0.001^**^

SMAS, Self-management Assessment Scale; QOL, quality of life. *p < 0.05; **p < 0.01.

a Independent-sample *t*-tests.

b Analyses of variance.

c Welch (W) method for approximate *F*-test.

### Descriptive statistics of measurement variables and their correlation analysis

The prevalence of frailty in middle-aged and older adult postoperative patients with gynecologic malignancies was 39.5%, of which no frailty accounted for 27.7%, vulnerable accounted for 32.8%, mild frailty accounted for 20.3%, moderate frailty accounted for 9.0%, severe frailty accounted for 10.2%. 43.8% of patients aged 45–59 years were vulnerable, 8.3% were mild frailty, and 1% were moderate frailty; 47.9, 10.4, and 4.2% of patients aged 60–69 years experienced mild, moderate, and severe frailty, in that order; and 20, 40, and 32% of patients aged 70–79 years experienced mild, moderate, and severe frailty, in that order; All eight patients ≥80 years old were severely frailty. The mean total scores for self-management, quality of life, and frailty were 125.81 ± 13.21, 69.26 ± 10.88, and 5.42 ± 2.48, respectively. Pearson correlation analysis showed that frailty was negatively correlated with self-management and quality of life, respectively (*r* = −0.774, *p* < 0.01; *r* = −0.654, *p* < 0.01), and self-management was positively correlated with quality of life (*r* = 0.624, *p* < 0.01). The frailty of postoperative patients with gynecological malignancies in different age groups is shown in Table 3. Descriptive statistics of the measurement variables and their correlation analyses are shown in Table 4.

**Table 3:** The frailty of postoperative patients with gynecological malignancies in different age groups.

Age,y	No frailty n (%)	Vulnerable n (%)	Mild frailty n (%)	Moderate frailty n (%)	Severe frailty n (%)
45–59	45(46.9)	42(43.8)	8(8.3)	1(1.0)	0(0.0)
60–69	4(8.3)	14(29.2)	23(47.9)	5(10.4)	2(4.2)
70–79	0(0.0)	2(8.0)	5(20.0)	10(40.0)	8(32.0)
≥80	0(0.0)	0(0.0)	0(0.0)	0(0.0)	8(100.0)

**Table 4:** Descriptive statistics of measurement variables and their correlation analysis.

Variables	Mean (SD)/n (%)	Self-management	General health	Frailty
Self-management	125.81 ± 13.21	1		
General health	69.26 ± 10.88	0.624^**^	1	
Frailty	5.42 ± 2.48	−0.774^**^	−0.654^**^	1
No frailty	49 (27.7)			
Vulnerable	58 (32.8)			
Mild frailty	36 (20.3)			
Moderate frailty	16 (9.0)			
Severe frailty	18 (10.2)			

**p < 0.01.

### Structural equation model

**Figure 2:**
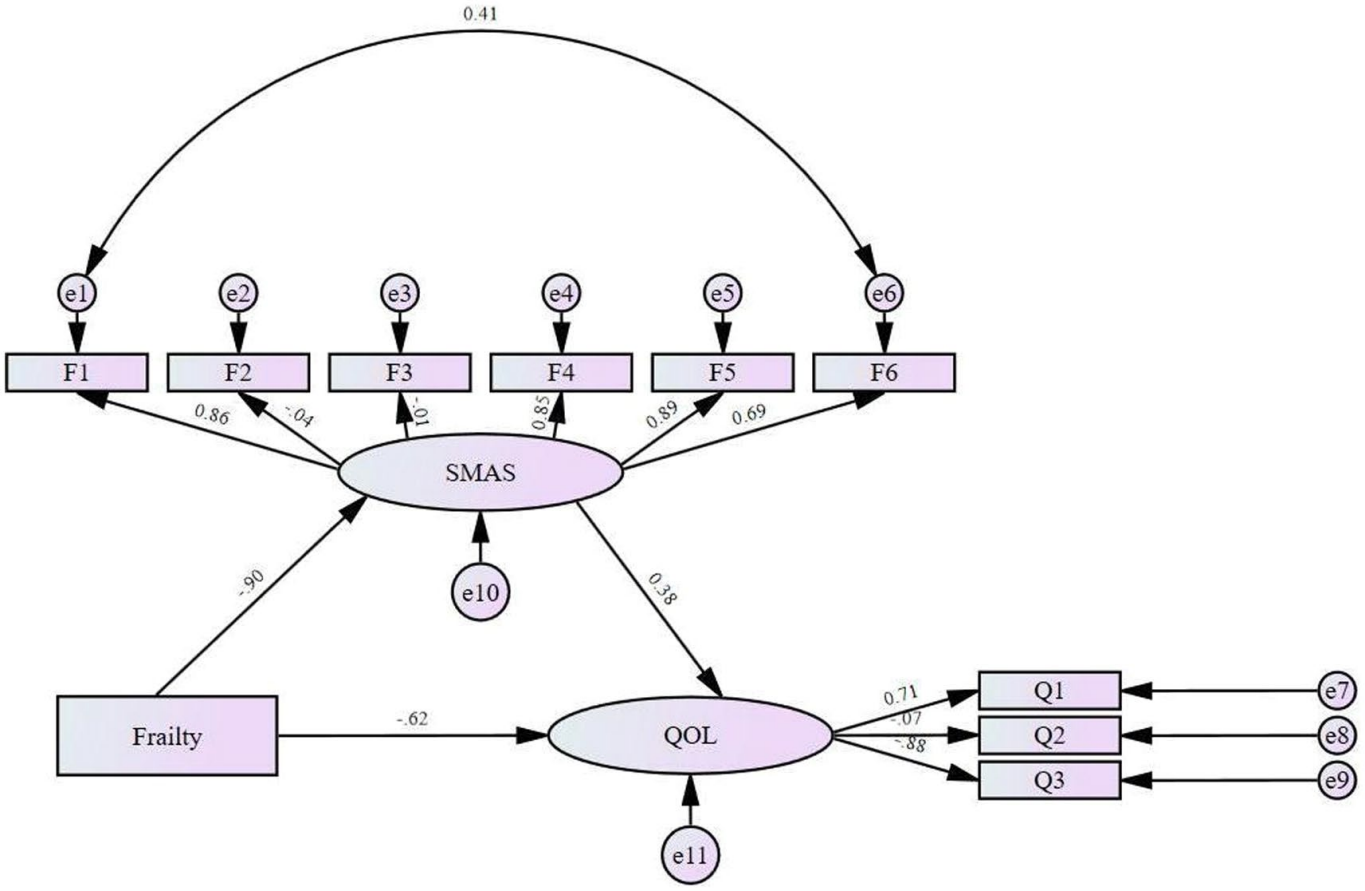
The modified model. SMAS, Self-management Assessment Scale; QOL, quality of life.

When the model is poorly fitted, the paths can be deleted or restricted, and new paths can be added according to the Modification Indices (MI) without violating the assumptions of the structural equation model. This process aims to improve the model fit and rationalize its structure. Initially, the model was tested for fitness, and it was found that some of the indicators did not meet the fitness parameter criteria, so the model was revised using the MI indicator adjustment method, which did not change the core assumptions or structure of the model. After adding the path relationship of “daily life management→self-efficacy” in this study, all the indexes reached the parameter standard, and the fitting effect was good, so the path was established. The modified model fit indicators are shown in Table 5. The modified model is shown in Figure 2.

**Table 5:** Modified model fit indicators.

Model	χ^2^/df	GFI	AGFI	NFI	CFI	RMSEA
Reference	<3	>0.9	>0.9	>0.9	>0.9	<0.08
Hypothetical	2.377	0.918	0.863	0.93	0.958	0.088
Modified	1.737	0.945	0.905	0.95	0.978	0.065

χ^2^/df, chi-square/degree of freedom; GFI, goodness-of-fit index; AGFI, adjusted goodness-of-fit index; NFI, normed fit index; CFI, comparative fit index; RMSEA, root mean square error of approximation.

The results of the structural equation model path relationship test are shown in Table 6, based on which it was concluded that frailty had a positive predictive effect on self-management (*β* = −0.902, *t* = −16.783, *p* < 0.001); self-management had a positive predictive effect on quality of life (*β* = 0.378, *t* = 3.071, *p* = 0.002); and frailty had a negative predictive effect on quality of life (*β* = − 0.619, *t* = −5.048, *p* < 0.001).

**Table 6:** Structural equation model path relationship test results.

Pathway			Nonstandardized coefficients	Standardized coefficients	S.E.	C.R.	P
SMAS	<−--	Frailty	−1.704	−0.902	0.102	−16.783	***
QOL	<−--	SMAS	0.62	0.378	0.202	3.071	0.002
QOL	<−--	Frailty	−1.919	−0.619	0.38	−5.048	***
F1	<−--	SMAS	1	0.863			
F2	<−--	SMAS	−0.021	−0.044	0.036	−0.57	0.569
F3	<−--	SMAS	−0.004	−0.007	0.049	−0.088	0.930
F4	<−--	SMAS	0.468	0.854	0.031	14.871	***
F5	<−--	SMAS	0.465	0.889	0.029	15.978	***
F6	<−--	SMAS	0.573	0.695	0.043	13.286	***
Q1	<−--	QOL	1	0.706			
Q2	<−--	QOL	−0.035	−0.069	0.039	−0.896	0.370
Q3	<−--	QOL	−1.572	−0.884	0.139	−11.328	***

SMAS, Self-management Assessment Scale; QOL, quality of life. ***p < 0.001.

The Bootstrap method was used to test the significance of the mediating effect. The results showed that the total effect of frailty on quality of life was −2.975 (95% CI: −3.386 ~ −2.534), the direct effect was −1.919 (95% CI: −2.836 ~ −1.054), and the mediating effect of self-management was −1.056 (95% CI: −2.045 ~ −0.207). Therefore, we believed that self-management played a partial mediating effect in the impact of frailty on quality of life, and the effect was significant, and the indirect effect accounted for 35% of the total effect. The results of the Bootstrap mediator effect test are shown in Table 7.

**Table 7:** Bootstrap mediating effect test results.

Parameter	Estimate	Lower	Upper	*P*	Relative effect
Indirect effects	−1.056	−2.045	−0.207	0.018	35%
Direct effects	−1.919	−2.836	−1.054	0.001	65%
Total effects	−2.975	−3.386	−2.534	0.001	–

## Discussion

This study used structural equation modeling to analyze the mediating pathways between frailty, self-management, and quality of life in middle-aged and older adult Chinese postoperative gynecologic malignancy patients, which helps to develop more targeted nursing interventions for patients.

As far as we know, this is the first report on the prevalence of frailty in middle-aged and older adult postoperative gynecological malignancy patients in China. Our study found frailty in 39.5% of patients, a rate that exceeds the 24.1% reported in the survey by Reiser et al. (40). This study used a frailty index to assess the degree of frailty in patients with gynecologic malignancies (including vulvar, endometrial, ovarian, or cervical cancer) who received initial treatment. Different assessment tools may result in differences in the degree of frailty. There are no standardized screening criteria for frailty in gynecological oncology. The EFS is a rapid, multidimensional assessment tool widely used for different types of cancer (41–43). It provides a comprehensive assessment of frailty and finely differentiates the degree of risk of frailty through 5 classification levels. Our findings showed that varying degrees of frailty was also present in patients aged 45–59 years. Therefore, the frailty assessment in patients with gynecological malignancies should not be based solely on actual age but on a multidimensional approach. The results of our study were slightly higher than those of other Chinese cancer patients (44). Since most of our participants were postmenopausal, the decline in ovarian function after menopause in women tends to lead to somatic aging. There is growing evidence that the hypothalamic–pituitary axis plays a potential role in regulating frailty (45). Rapid growth and extensive infiltration of malignant tumors accelerate the occurrence and development of frailty, which leads to changes in the physiological and psychological status of patients and seriously affects the quality of life. This suggests that finding potential relationship variables between frailty and quality of life is significant.

Our model showed that frailty had a direct impact on quality of life and also indirectly affected quality of life through self-management. Frailty was significantly negatively correlated with quality of life, consistent with previous studies (46). This suggests that managing frailty can improve quality of life. First of all, physical and psychological functions are the public part of assessing frailty and quality of life, which helps to explain that frailty is an important factor affecting quality of life (47). Secondly, frailty often progresses in tandem with various chronic diseases (48), which may lead to a decline in bodily functioning, among other things, poor health conditions exacerbate the deterioration of quality of life, which can be used as a potential explanation for the relationship between frailty and poorer quality of life. However, frail cancer patients’ awareness of improving their quality of life is no different from, or even lower than, that of non-frail people (49). So helping cancer patients to develop a concept of active health management and teaching them self-monitoring is a clinically necessary initiative.

We found that self-management had a partial mediating effect between frailty and quality of life, suggesting that individuals with good self-management skills and no frailty may have a higher quality of life. The chronicity of cancer means that anti-cancer is a protracted war, and lengthy treatments can negatively affect the patient’s emotional state and self-efficacy, ultimately leading to a deterioration in the quality of life (50). Different types of surgeries vary in terms of procedure, level of trauma, and recovery cycle, and these differences may affect a cancer patient’s postoperative recovery (51). Studies have established that regular, evidence-based, individualized exercise workouts and maintaining good nutritional status are key self-management strategies for preventing frailty (52). Therefore, clinical professionals can organize face-to-face or online sessions that provide medical education about frailty, goal-setting, problem-solving, exercise training, nutritional counseling, and guidance on emotional management. Patients are provided with a written treatment manual, and group exercises are conducted at the end of each session to reinforce the concepts learned. Also, clinicians can utilize the intelligent app to track exercise report history and connect to a self-monitoring web platform. In addition, clinical staff can provide self-management support as patients independently develop goals and action plans. These are essential measures to develop the self-management skills of frail patients. There is growing evidence that high-quality self-management support is needed in cancer care (53). First, support from family, friends, doctors, social workers, and other roles helps to help patients face the challenges of the disease more positively (54). Second, the individual’s own self-cognition is also a factor that influences the patient’s ability to self-manage (55). Third, according to self-efficacy theory, negative emotions lead to low self-management ability (56). In conclusion, it is very important to provide more comprehensive and professional medical services, conduct timely emotional assessments, establish a good support system, and encourage patients to explore science in order to improve their self-management ability and quality of life.

Our study found that postoperative gynecological malignancy patients between 45 and 59 were mostly vulnerable. In contrast, patients over 60 years of age were more have mild or severe frailty. This may be related to diminished physiologic function and accumulation of chronic disease. These groups may require different self-management strategies and medical support. For example, patients aged 45–59 years focus on preventive treatments to minimize the onset of debilitation. This includes acquiring health monitoring and disease self-management skills, actively coping with emotional challenges, and maintaining a healthy lifestyle. In contrast, patients over 60 require more aggressive treatment and care. This includes regular medical follow-up, personalized medication, and rehabilitation programs. In addition, given that patients may have multiple medical conditions, an interdisciplinary team is needed to provide integrated and comprehensive care. Therefore, providing personalized, multilevel care for middle-aged and older adult postoperative gynecologic malignancy patients is essential.

This study added to the understanding of frailty in middle-aged and older adult postoperative gynecologic malignancy patients. It elucidates that frailty improves quality of life through self-management. Based on these findings, we consider the implications for practice. Clinical professionals should actively screen for early frailty and implement interventions targeting self-management to prevent or minimize adverse health outcomes, especially in middle-aged and older patients. In future theoretical studies, we will conduct subgroup analyses of this population to provide insight into the impact of frailty on quality of life. More research on multilevel interventions is recommended. It is recommended that policymakers consider incorporating self-management skills into long-term care programs for cancer patients and provide the necessary resources and support to help patients better manage their health. In addition, timely identification and intervention of frailty can reduce the consumption of healthcare resources.

This study has several limitations. First, it was only a single-center data analysis, and it may not be possible to extend the results to all patients with gynecologic malignancies. Second, this was a cross-sectional study, which made it difficult to determine whether there was a causal relationship between the variables. Subsequent researchers can conduct longitudinal or qualitative studies between relevant variables for a comprehensive and in-depth analysis. Finally, subgroup analysis of sufficient samples is necessary, considering that significant effects may vary by surgical procedure and tumor type.

In summary, in this study, frailty in middle-aged and older adult postoperative gynecological malignancy patients has a direct impact on quality of life. It can also indirectly impact the quality of life through the mediating role of self-management. There is a need to incorporate self-management support interventions into care programs for middle-aged and older adult patients with gynecological malignancies in the future to promote better patient health.

## Data Availability

The original contributions presented in the study are included in the article/supplementary material, further inquiries can be directed to the corresponding authors.
